# Safety and Efficacy of Carotid Artery Stenting with the CGuard Double-layer Stent in Acute Ischemic Stroke

**DOI:** 10.1007/s00062-022-01209-3

**Published:** 2022-09-07

**Authors:** Tomas Klail, Christoph Kurmann, Johannes Kaesmacher, Adnan Mujanovic, Eike I. Piechowiak, Tomas Dobrocky, Sara Pilgram-Pastor, Adrian Scutelnic, Mirjam R. Heldner, Jan Gralla, Pasquale Mordasini

**Affiliations:** 1 University Institute of Diagnostic and Interventional Neuroradiology, Inselspital, Bern University Hospital, University of Bern, Bern, Switzerland; 2 Department of Neurology, Inselspital, Bern University Hospital, University of Bern, Bern, Switzerland; 3 University Institute of Diagnostic and Interventional and Pediatric Radiology, Inselspital, Bern University Hospital, University of Bern, Bern, Switzerland

**Keywords:** Emergency, Recanalization, Tandem occlusion, Stenosis, Embolism

## Abstract

**Background:**

Double-layer stents show promising results in preventing periinterventional and postinterventional embolic events in elective settings of carotid artery stenting (CAS). We report a single-center experience with the CGuard stent in the treatment of acute ischemic stroke (AIS) due to symptomatic internal carotid artery (ICA) stenosis or occlusion with or without intracranial occlusion.

**Methods:**

We retrospectively analyzed all patients who received a CGuard stent in the setting of AIS at our institution. Neuroimaging and clinical data were analyzed with the following primary endpoints: technical feasibility, acute and delayed stent occlusion or thrombosis, distal embolism, symptomatic intracranial hemorrhage (sICH) and functional outcome at 3 months.

**Results:**

In 33 patients, stenting with the CGuard was performed. Stent deployment was successful in all patients (28 with tandem occlusions, 5 with isolated ICA occlusion). Transient acute in-stent thrombus formation occurred in three patients (9%) without early stent occlusion. Delayed, asymptomatic stent occlusion was seen in 1 patient (3%) after 49 days. Asymptomatic periinterventional distal emboli occurred in 2 patients (6%), 1 patient experienced a transient ischemic attack 79 days after the procedure and 1 patient (3%) developed sICH. Favorable clinical outcome (mRS 0–2) at 3 months was achieved in 12 patients (36%) and the mortality rate was 24%.

**Conclusion:**

The CGuard use in emergencies was technically feasible, the safety has to be confirmed by further multicentric studies.

## Introduction

Several studies have shown that carotid artery stenting (CAS) is a valid treatment option for internal carotid artery (ICA) stenosis [1–3]; however, a potential complication of CAS is periprocedural or delayed embolism either due to dislodgement of plaque debris during periprocedural manipulation or, especially in open-cell stents, due to protrusion of plaque or thrombotic material through the struts of the expanded stent [4, 5].

To address this issue, a new generation of double-layered stents has been introduced into clinical practice. Studies have demonstrated a reduction of procedure-related embolizations in the elective setting in patients on standard double antiplatelet therapy [6, 7]. These stent features would also be useful in acute stroke treatment since most plaques are vulnerable to adherent thrombus and often prone to embolization. This could be prevented by containing the thrombus between the arterial wall and the outer layer of the stent.

However, the debate around open versus closed cell stents has not yet been settled since many metal braided double-layer stents seem to show a tendency to in-stent thrombus formation or decreased vessel compliance in the acute setting [8–11]. The CGuard stent (InspireMD Inc., Tel Aviv, Israel) represents a new generation of carotid double-layered stents [12] comprised of an inner open-cell laser-cut nitinol stent and an outer closed-cell polyethylene terephthalate mesh layer, thus reducing the amount of thrombogenic material in the stent [13].

We therefore hypothesized that the CGuard stent would be suitable for application in emergency CAS and we present the first analytic overview of its use in acute extracranial ICA stenting.

## Methodology

### Patient Selection

All patients with acute symptomatic extracranial ICA occlusion or high-grade stenosis with or without intracranial vessel occlusion (tandem occlusion), who were treated with the CGuard stent at the University Hospital of Bern, Switzerland, were collected prospectively between December 2018 and November 2021. The study was approved by the local ethics committee (amendment access number: 231/2014).

Patients underwent endovascular intervention immediately after computer tomography (CT) or magnetic resonance imaging (MRI) if (1) diagnosis of ischemic stroke was established on imaging by a neuroradiologist, (2) intracranial hemorrhage was excluded on CT or MRI, (3) symptom duration was not longer than 24 h, (4) no further clinical conditions contraindicated the procedure [14] and (5) occlusion or severe stenosis of the cervical ICA (with or without an intracranial occlusion) was demonstrated by initial or peri-interventional imaging. When indicated, intravenous thrombolysis (IVT) was administered prior to endovascular therapy.

### Device

The CGuard stent is a double-layered stent consisting of an inner open-cell laser-cut nitinol stent and an outer closed-cell polyethylene terephthalate mesh layer with filament diameters of 92–125 µm and 25 µm, respectively. It is a self-expanding system delivered through a 6 French access catheter and is available in sizes of 6–10 mm in diameter and 20–60 mm in length [15]. The pore size of the inner layer when fully expanded is 150–180 µm [6].

### Endovascular Procedure

The general approach to treating tandem occlusions at our institution has been described previously [16, 17]. Briefly, all patients underwent the endovascular procedures under general anesthesia. After puncture of the common femoral artery, an 8 or 9 French sheath was introduced. Selective digital subtraction angiography (DSA) of the three non-occluded cervical vessels was routinely performed on a biplane high-resolution angiographic system (Artis Icono and Artis Q; Siemens, Erlangen, Germany) to assess cross flow and collaterals, followed by angiography of the occluded vessel. Based on clinical data and angiographic morphology, atheromatous occlusions were distinguished from ICA dissections.

A retrograde approach was chosen for all patients with tandem occlusions (treatment of the extracranial ICA occlusion or stenosis after intracranial recanalization) [3, 18]. After placement of a guiding catheter (8 French Guider Softtip, Boston Scientific, Marlborough, MA, USA; 9 French Merci balloon-guide catheter, Concentric Medical, Mountain View, CA, USA) a 0.021″ microcatheter (Prowler Select Plus, Codman & Shurtleff Inc., Raynham, MA, USA; Phenom 21, Medtronic, Dublin, Ireland) was navigated through the stenosis over a 0.014″ microwire (Traxess, Microvention, Aliso Viejo, CA, USA) under proximal balloon occlusion and flow arrest. Only when intracranial access was not possible due to difficult vessel anatomy or high-grade stenosis, was balloon angioplasty of the ICA stenosis performed first. Once the microcatheter position distally to the occlusion or stenosis had been confirmed by a contrast injection, an intermediate 5 or 6 French catheter (5max ACE, Penumbra, Alameda, CA, USA; Vasco +35Aspi, Balt Extrusion, Montmorency, France; Sophia 5/6 French, Microvention, React 68/71, Medtronic) was advanced over the microcatheter into the distal ICA. The intracranial thrombus was passed with a microcatheter over the microwire and mechanical thrombectomy (MT) was performed using a stent retriever device with distal aspiration. Successful recanalization was defined as a modified thrombectomy in cerebral infarction (mTICI) of 2b or 3.

Before performing cervical stenting or PTA, a filter protection device (FilterWire EZ, Boston Scientific) was placed distal to the ICA occlusion to protect against distal embolization before deflation of the balloon-guided catheter and implantation of the CGuard stent at the stenotic site of the ICA. The final decision on whether to stent was at the discretion of the treating physicians. Predilatation and postdilatation was performed using balloon angioplasty (Aviator Plus PTA Dilatation Catheter, Cardinal Health, Dublin, OH, USA). DSA was performed at the end of the stent implantation process to check for acute in-stent thrombosis, distal embolism or other complications. Standard antiplatelet therapy consisted of intravenous administration of 250–500 mg aspirin after intracranial recanalization, prior to stenting. Control imaging was performed within 24 h to exclude intracranial hemorrhage and assess infarct evolution. If no intracranial hemorrhage was detected, dual antiplatelet therapy (DAPT) with additional clopidogrel 75 mg was initiated and continued for 3–6 months. All imaging data were analyzed by an interventional neuroradiologist.

### Outcome Evaluation

The National Institutes of Health Stroke Scale (NIHSS) was assessed by a neurologist on admission and at discharge. Following internal guidelines, CT or MRI within 24 h after the intervention was used to assess intracranial bleeding and infarct evolution. Symptomatic intracranial hemorrhage (sICH) was defined as hemorrhage associated with a decline of ≥ 4 points in the NIHSS [19]. Stent patency was assessed using ultrasound or CT or MR angiography. Routine clinical follow-up was performed at 3 months by an independent neurologist to evaluate patients’ recovery. The modified Rankin Scale (mRS) at 3 months was used as the indicator of clinical outcome.

### Statistical Analysis

All characteristics (baseline, procedure, outcome) were analyzed using descriptive statistics. Normally distributed continuous variables are presented as mean ± standard deviation (SD), non-normally distributed continuous variables as median with interquartile range (IQR). Categorical variables are expressed as absolute numbers and percentages or as median with IQR. Normality was tested using graphical distribution and the Shapiro-Wilk test. Missing values were not imputed. All calculations were performed using R (R Core Team, Version 4.0.0) [20].

## Results

### Patient Population

Of the 33 patients, 25 (76%) were male, mean age was 73 years (IQR 63–80 years) and the median NIHSS score on admission was 13 (IQR 11–20). Of the patients 11 (33%) were under antiplatelet or anticoagulation therapy at the time of admission: 9 on aspirin monotherapy, 1 on clopidogrel and 1 on edoxaban.

**Fig. 1:**
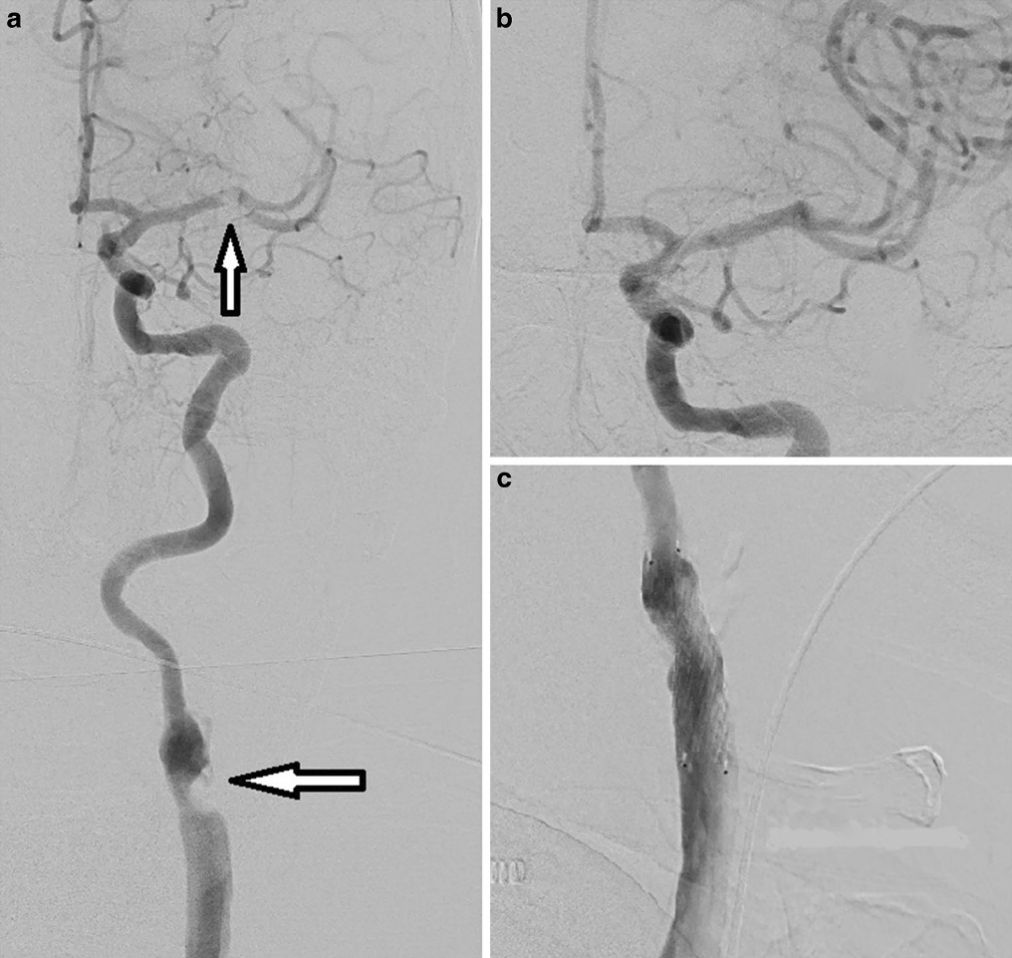
Patient with an internal carotid artery (ICA) stenosis on the left side and a concurrent distal tandem occlusion. **a** Diagnostic angiogram showing an occlusion in the M1 segment (*smaller arrow*) of the middle cerebral artery (MCA) and a high-grade stenosis at the carotid bifurcation (*larger arrow*). **b** Control angiogram after mechanical thrombectomy shows reperfusion of the MCA. **c** Control angiogram after CGuard stent placement shows normal caliber of the ICA

The median Alberta Stroke Programme Early CT Score (ASPECTS) on emergency imaging was 7 (IQR 5–8). Of the patients 28 presented with tandem occlusion stroke, most commonly affecting the M1 segments (13/23) and M2 segments (12/23) of the middle cerebral artery (MCA), and the terminal ICA (5/23) (Fig. 1). Three patients also had a second intracranial occlusion in the M2, M3, or anterior cerebral artery (Table 1).

**Table 1:** Patient baseline characteristics

	Overall
*Age years (median [IQR])*	73 [63–80]
*Male, n (%)*	25 (76)
*NIHSS on admission (median [IQR]) (n* *=* *25/27)*	13 [11–20]
*Pre-interventional antiplatelet medication, n (%)*	
None	22 (67)
Aspirin	9 (27)
Clopidogrel	1 (3)
Edoxaban	1 (3)
*Intracranial occluded vessel, n (%)*	
ICA	5 (15)
M1	13 (38)
M2	12 (35)
M3	2 (6)
M4 ACA	1 (3) 1 (3)
*Emergency imaging modality, n (%)*	
CT	14 (42)
MRI	14 (42)
CT and MRI	5 (19)
*ASPECTS on admission (median [IQR])*	7 [5–8]
*Intravenous thrombolysis, n (%)*	13 (39)
Bridging therapy	11 (33)
*Risk factors, n (%)*	
Atrial fibrillation	3 (9)
Diabetes mellitus	7 (21)
Hypertension	20 (61)
Dyslipidemia	19 (58)
Smoking	12 (36)
Coronary heart disease	3 (9)
Previous stroke	1 (3)
Previous TIA	1 (3)

*ASPECTS* Alberta Stroke Programme early CT score, *ICA* internal carotid artery, *IQR* interquartile range, *M1–4* middle cerebral artery segments, *NIHSS* National Institutes of Health Stroke Scale, *TIA* transient ischemic attack

Of the 33 patients, 28 (85%) underwent MT of the intracranial occlusion prior to stenting of the extracranial ICA with the CGuard stent. In 5 patients (15%), only stenting of the cervical ICA was performed, IVT was performed in 13 (39%) patients, 11 underwent subsequent MT of an intracranial occlusion and 1 received IVT without MT (drip-and-ship patient with spontaneous recanalization of intracranial occlusion). One patient received intra-arterial urokinase along with MT as a rescue therapy.

### Intervention

The underlying etiology of the ICA occlusion or stenosis was atherosclerosis in 29 (88%), ICA dissection in 3 patients (6%), and unclear in 1 patient (3%). Median time from symptom onset to recanalization was 371 min (IQR 270–510 min). Final intracranial recanalization success was mTICI2b in 11/28 patients (39%) and mTICI3 in 17/28 patients (61%). After deployment, 82% of stents were dilated with PTA and good recanalization was achieved in all patients. All but one patient received a peri-interventional dose of 250–500 mg aspirin (one patient had a procedural intracranial hemorrhage determined by flat panel CT). One day after the intervention, DAPT with aspirin and clopidogrel was initiated in 25 patients, while 5 remained on aspirin monotherapy. Due to death shortly after the intervention, three patients received no therapy. Interventional characteristics are summarized in Table 2.

**Table 2:** Interventional characteristics

	Overall
*Time from symptom onset or last seen well to recanalization in minutes (median [IQR])*	371 [270–510]
*Procedure time in minutes (median [IQR])*	50 [35–70]
*Final mTICI*^*a*^*, n (%)*	
2b	11 (39)
3	17 (61)
*ICA lesion type, n (%)*	
Large artery atherosclerosis	29 (88)
Dissection	3 (9)
Unknown	1 (3)
*Size of stent (diameter in mm), n (%)*	
4	1 (3)
6	1 (3)
7	13 (39)
8	11 (33)
9	4 (12)
10	3 (9)
*Size of stent (length in mm), n (%)*	
30	5 (15)
40	26 (79)
60	2 (6)
*PTA, n (%)*	27 (82)
*CGuard stent patency at the end of procedure, n (%)*	33 (100)
*Additional intracranial stenting, n (%)*	1 (3)
*Complications, n (%)*	
Dissection related to mechanical thrombectomy Dissection related to ICA stenting	3 (9) 1 (3)
Distal embolism in new territory	2 (6)
Distal embolism in target downstream territory	2 (6)
Acute in-stent thrombus formation	3 (9)

*CT* computed tomography, *ICA* internal carotid artery, *IQR* interquartile range, *MRI* magnetic resonance imaging, *mTICI* modified thrombolysis in cerebral infarction, *PTA* percutaneous transluminal angioplasty

^a^After intracranial recanalization. Excluding 5 patients who did not undergo mechanical thrombectomy

### Procedural Complications

Acute in-stent thrombus formation occurred in three patients (9%, 3/33). After PTA and aspiration (*n* = 1), aspiration alone (*n* = 1) or no rescue maneuver (due to only minor and peripheral thrombus, *n* = 1), all stents were patent at the end of the intervention. Glycoprotein IIb/IIIa inhibitors or heparin were not administered in these cases. Iatrogenic arterial dissection occurred in four patients (12%), only one dissection occurred after CAS with CGuard, three occurred while gaining intracranial access for mechanical thrombectomy by advancing the guide/intermediate catheter through due to tortuous vessel anatomy. After stenting with the CGuard, there was a suspected small dissection in one case at the distal end of the stent without hemodynamic relevance, and further treatment was not deemed necessary. Of the other three cases, two involved the cervical part of the ICA and which required additional stenting and extension of the lesion to the supraophthalmic segment without hemodynamic relevance occurred in one patient. In three of them, only the cervical part of the ICA was involved and extension of the lesion to the supraophthalmic segment occurred in one patient. Two dissections were flow-limiting and required additional stenting. Distal embolisms were found in the infarct territory on final control DSA after stenting in two patients (6%) and new clinically asymptomatic infarcts in new territory in follow-up imaging in two other patients (6%).

### Clinical Follow-up

Median NIHSS at discharge was 7 (IQR 2–15). All patients with available early follow-up imaging (CT, MRI or ultrasound) showed patent stents (31/33; 1 patient died due to sICH and 1 patient was transferred to another hospital before follow-up examination). A sICH occurred in one patient (3%) and asymptomatic intracranial hemorrhage (asICH) in nine patients (27%). Of the nine patients with aICH, seven were due to hemorrhagic transformation (five patients with hemorrhagic infarction (HI) 1, one with HI2 and two with parenchymal hematoma (PH) 2) and two due to SAH. Median duration of follow-up after the procedure was 90 days (IQR 89–98). Postinterventional stent occlusion occurred in one patient after 49 days, 1 patient suffered a transient ischemic attack (3%) and 12 patients (36%) had a follow-up mRS ≤ 2 at 3 months. Patients with unfavorable outcome (defined as 3‑month mRS > 2) presented with significantly higher age (79 vs. 66 years, *p* = 0.044) and a tendency towards lower ASPECTS score (7 vs. 8, *p* = 0.149) The median NIHSS at 3 months in all survivors was 1 (IQR 0–4). The mortality rate at 3 months was 24% (8 patients). All follow-up characteristics are shown in Table 3.

**Table 3:** Outcome characteristics

	Overall	Missing (*n*)
*NIHSS on discharge (median [IQR])*	7 [3–13]	2
*Bleeding complications, n (%)*		
sICH	1 (3)	0
asICH	9 (27)	0
*New infarct on 24* *h follow-up imaging, n (%)*	2 (6)	0
*Stent patency in survivors at 24–48* *h, n (%)*	31 (100)	2
*Stent occlusions in long-term follow-up, n (%)*	1 (3)	7
*Functional outcome*		
NIHSS at 3 months in survivors (median [IQR])	1 [0–4]	7
Symptomatic stroke within 3 months, *n* (%)	0 (0)	3
mRS score at 3 months (median [IQR])	3 [2–6]	3
mRS ≤ 2 at 3 months, *n* (%)	12 (36)	3
Mortality at 3 months, *n* (%)	8 (24)	3

*IQR* interquartile range, *NIHSS* National Institutes of Health Stroke Scale, *sICH* symptomatic intracranial hemorrhage, *asICH* asymptomatic intracranial hemorrhage, *mRS* modified Rankin Scale

## Discussion

The main findings of our study are: 1) The rate of acute in-stent thrombosis of the CGuard stent using a single antiplatelet protocol with aspirin is low in the acute stroke setting, 2) the use of the CGuard stent in the acute setting results in high patency rates at follow-up, and 3) periprocedural and postprocedural thromboembolic events after deployment of the CGuard stent are rare.

The finding that single-layer closed-cell stents were associated with lower rates of thromboembolic events in patients undergoing CAS compared to open-cell stents led to the introduction of double-layer carotid artery stents. The main objective was to prevent dislodgement of debris and subsequent embolization into the intracranial vessels while maintaining stent flexibility [21]. Their superior qualities have been confirmed by preliminary clinical studies [6, 22]; however, the clinical results from emergency settings remain conflicting as higher rates of in-stent thrombus formation were observed in braided metal double-layered stents with more thrombogenic material [8, 9]. Use of the CGuard stent, which has less thrombogenic material, has led to very low rates of postprocedural thrombotic events in elective settings; however, it was unclear whether these results can be translated to emergency ICA stenting.

### Acute and Delayed In-stent Thrombus Formation

Dual antiplatelet therapy (DAPT) has been established as standard therapy for the prevention of in-stent thrombus formation [23]. Therapy duration ranges from 3 to 6 months and is a trade-off between in-stent thrombosis prevention and increased risk for sICH. Of our patients 25 (76%) received DAPT, the others either had a contraindication (e.g. hemorrhage on early follow-up imaging) or died before DAPT could be initiated.

The rate of acute in-stent thrombus formation in our cohort was 9%. This number is considerably lower than that reported by Bartolini et al. [9] (52.4%) or Pfaff et al. [24] (20.8%), who mainly examined the Casper-RX stent (MicroVention) in the acute setting. One explanation could be the different composition of the CGuard and the Casper-RX stent; the latter has a braided nitinol double-layer with potentially higher thrombogenicity. Yilmaz et al. [8], who examined a wider range of stents in acute tandem occlusion settings, also reported a much higher thrombosis rate (50%); however, our numbers are consistent with a study by Eker et al. who found a 9% [17] in-stent thrombosis rate in acute settings using both double-layer and single-layer stents for CAS in patients with tandem occlusions.

After adequate rescue therapy, all stents in our cohort were patent at the end of the intervention as well as at the 24–48 h follow-up examination. These results are even better than those generally reported for CAS in tandem occlusions. For example, in the TITAN study [25] the acute in-stent thrombosis rates during the intervention or within 24 h after the intervention were 4% and 15% in the antiplatelet pretreated and non-pretreated groups, respectively. Wallocha et al. [26] reported a 5% ICA re-occlusion rate at 24 h in a study using single-layer stents with postprocedural aspirin or DAPT. Another study on double-layered stents, CGuard and Roadsaver (Terumo, Shibuya, Japan), by de Vries et al. [21] observed postprocedural stent occlusions in five patients (9%). None of the affected patients was on antiplatelet therapy at time of presentation and 4/5 were treated with Roadsaver.

During the 3‑month follow-up period, 1/17 patients (6%) had a delayed stent occlusion (after 49 days). This patient was asymptomatic and on standard DAPT. The stent occlusion was discovered incidentally during a routine ultrasound follow-up examination. In 7 patients, 3‑month follow-up examination of stent patency was missing either due to imaging in other hospitals or due to postponed visits related to the COVID-19 pandemic. Our rate of delayed stent thrombosis is lower than in the study by Lamanna et al. [27] who evaluated deployment of the Casper-RX stent in the acute setting and reported 1/19 patients with delayed in-stent thrombosis (1 month after the intervention). In contrast, studies evaluating the CGuard stent in the elective setting found no re-occlusions after 30 days [22, 28] or even after 12 months [29]. CT or MRI at 3 months was unavailable in 9 patients, making it impossible to assess the occurrence of silent stroke in these patients.

### Other Procedure-related Complications and Clinical Outcome

The rate of postinterventional symptomatic stroke in our cohort (0%) is in line with previous studies evaluating the CGuard in the elective setting. Whereas the PARADIGM study observed one event (1%) [30] IRON-GUARD [29] and the study by Wissgott et al. [22] reported no events; however, one patient (3%) had new neurologic symptoms (TIA after 97 days). sICH was observed in one patient who subsequently died, which is a lower rate than in other comparable studies [17, 27].

The 3‑month mortality rate was 24%, comparable to another multicenter study assessing acute stenting in tandem occlusion with various stents (24%) [17] but higher than studies evaluating the Casper-RX stent (10% [27] and 14.3% [9], respectively) or in the TITAN study (11% and 19%) [25]. Patients with poor outcome were more likely to be older. There was also a non-significant tendency towards lower ASPECTS score on admission. Other baseline or procedural characteristics did not result statistically significant.

### Clinical Implications

Deployment of the CGuard stent in the acute setting was technically feasible and successful in all patients, despite its relative stiffness when compared to other double-layered stents [22]. Furthermore, the low rate of acute and delayed in-stent thrombus formation and of other procedure-related complications (e.g., distal emboli with new infarcts) as well as the low rate of occlusion of the stent in the follow-up period supported the effectiveness and safety of using the CGuard stent in the acute setting.

### Strengths and Limitations

To our knowledge, this is the first study analyzing the use of the CGuard double-layered stent in emergency interventions.

The limitations of this study are: (1) its nonrandomized, single-center design, in which patients were prospectively entered into the Swiss Stroke Registry and then retrospectively analyzed, (2) the non-consecutive inclusion of patients, (3) the relatively small number of treated patients, which could have led to bias and lower generalizability of the results, (4) the absence of a matched comparison group, (5) a relatively large number of patients who were lost to follow-up due to examinations in other hospitals, and (6) non-standardized antiplatelet regimen after the intervention and the making of therapy decisions on an individual basis. Ideally, randomized controlled studies would be needed for confirmation of our results, or as alternatives, cohort-studies with matched controls or the inclusion of patients into multi-centric quality assurance registries.

### Conclusions

The CGuard use in emergencies was technically feasible, the safety has to be confirmed by further multicentric studies.
